# Building a Data Platform for Cross-Country Urban Health Studies: the SALURBAL Study

**DOI:** 10.1007/s11524-018-00326-0

**Published:** 2018-11-21

**Authors:** D. Alex Quistberg, Ana V. Diez Roux, Usama Bilal, Kari Moore, Ana Ortigoza, Daniel A. Rodriguez, Olga L. Sarmiento, Patricia Frenz, Amélia Augusta Friche, Waleska Teixeira Caiaffa, Alejandra Vives, J. Jaime Miranda, the SALURBAL Group

**Affiliations:** 1 Urban Health Collaborative, Drexel University, Philadelphia, PA USA; 2 Nesbitt Hall, 3215 Market St, 2nd Floor, Philadelphia, PA 19104 USA; 3 Department of City & Regional Planning, University of California - Berkeley, Berkeley, CA USA; 4 Department of Epidemiology, Universidad de los Andes, Bogota, Colombia; 5 School of Public Health, Universidad de Chile, Santiago, Chile; 6 Departament of Preventive and Social Medicine, Universidade Federal de Minas Gerais, Belo Horizonte, Brasil; 7 School of Medicine, Pontifica Universidad Católica de Chile, Santiago, Chile; 8 School of Medicine, Universidad Peruana Cayetano Heredia, Lima, Peru

**Keywords:** Urban health, Latin America, Cities, Built environment, Social Environment, Multilevel Models, Mortality, Health Survey

## Abstract

Studies examining urban health and the environment must ensure comparability of measures across cities and countries. We describe a data platform and process that integrates health outcomes together with physical and social environment data to examine multilevel aspects of health across cities in 11 Latin American countries. We used two complementary sources to identify cities with ≥ 100,000 inhabitants as of 2010 in Argentina, Brazil, Chile, Colombia, Costa Rica, El Salvador, Guatemala, Mexico, Nicaragua, Panama, and Peru. We defined cities in three ways: administratively, quantitatively from satellite imagery, and based on country-defined metropolitan areas. In addition to “cities,” we identified sub-city units and smaller neighborhoods within them using census hierarchies. Selected physical environment (e.g., urban form, air pollution and transport) and social environment (e.g., income, education, safety) data were compiled for cities, sub-city units, and neighborhoods whenever possible using a range of sources. Harmonized mortality and health survey data were linked to city and sub-city units. Finer georeferencing is underway. We identified 371 cities and 1436 sub-city units in the 11 countries. The median city population was 234,553 inhabitants (IQR 141,942; 500,398). The systematic organization of cities, the initial task of this platform, was accomplished and further ongoing developments include the harmonization of mortality and survey measures using available sources for between country comparisons. A range of physical and social environment indicators can be created using available data. The flexible multilevel data structure accommodates heterogeneity in the data available and allows for varied multilevel research questions related to the associations of physical and social environment variables with variability in health outcomes within and across cities. The creation of such data platforms holds great promise to support researching with greater granularity the field of urban health in Latin America as well as serving as a resource for the evaluation of policies oriented to improve the health and environmental sustainability of cities.

## Introduction

By 2050, at least 70% of the world’s population will live in cities [1]. Urban policies impact important determinants of health, health equity, and environmental sustainability [2]. However, there is limited empirical evidence on what factors may make some cities healthier, more equitable, or more environmentally sustainable than others [3–6]. Latin America, with over 80% of its population living in urban areas [1] and a diversity of geographies and socioeconomic circumstances, presents a unique opportunity to study the impacts of urban living on health.

Cities in Latin America are heterogeneous in size; have diverse physical, social, and economic environments; and are frequently characterized by large social inequalities [3, 7]. Cities of the region have also generated innovations in transportation, urban redevelopment, food policies, and social programs [8–12]. The SALURBAL (*Salud Urbana en America Latina*/Urban Health in Latin America) project launched in 2017 aims to leverage the heterogeneity and innovation observed across Latin American cities to study drivers of urban health, health equity, and environmental sustainability in order to inform urban policies worldwide [13].

A critical need in any cross-city comparison study is the creation of a data platform that can support between- and within-city comparisons and that can be flexibly linked to various types of data defined at different levels of aggregation [14–17]. In this paper, we (1) describe the design of the SALURBAL data structure, including how cities are operationalized; (2) summarize the approach to obtaining and harmonizing health data; (3) describe priority social and physical environment indicators; (4) provide examples of how the data structure can be used to answer meaningful research questions about within and between-city variation in health; and (5) discuss selected challenges in creating this resource. Our goal is to inform similar data compilation efforts in other regions in order to enhance the ability to understand drivers of urban health and the impact of various urban policies on health.

## A Flexible Multilevel Data Structure

Conducting within-city and cross-city comparisons of urban health necessitates: (1) identifying the universe of “cities”; (2) operationalizing cities and geographic subunits within cities including neighborhoods in ways that permit linkages to available health and environmental data; (3) obtaining, processing, and harmonizing health data as well as data on social and physical environments; and (4) integrating all available information within a multilevel data structure that allows definition and measurement of constructs and investigation of questions at different levels. SALURBAL developed a data structure that accommodates information available for different geographic units and allows for heterogeneity, both geographically and over time. The process was guided by the principle that pragmatic albeit imperfect geographic definitions would be necessary to advance the project and that these definitions could be refined as the project progresses. The data structure developed allows for complementary analytical approaches that may be used to varying extents as the project evolves.

### Identifying and Operationalizing Cities

There is no unique way to define a city, but there are at least three possible types of definitions: (1) administrative definitions based on political or administrative boundaries; (2) definitions based on social or economic functions, such as country-defined metropolitan areas, that capture interconnectedness between a core city and nearby areas; and (3) definitions based on the geographic extent of urban areas identified from satellite imagery using standardized criteria [14–16, 18–20].

An advantage of administrative definitions of cities is that they can be linked to administrative and political responsibility and are often easy to link to health data. A disadvantage is that in large urban areas administratively defined cities often only capture a core city and may not fully represent the entire urban agglomeration. [21, 22]

Functional definitions such as metropolitan areas better capture the urban agglomeration around administratively defined core cities and have the important advantage of being based on social and economic relations between the core city and its surrounding areas. There are two broad types of functional definitions for these agglomerations. A first definition is based on networks, like water or road networks, while the second definition is based on travel patterns, which define labor or commute areas that are economically linked. Functional definitions receive a variety of names across different countries (e.g., metropolitan areas or urban agglomerations). Considerations of these broader geographic areas may be important to understand the drivers of urban health and the impact of urban health policies. However, these areas are defined using different criteria in different countries making cross-country comparisons difficult and may in some cases include surrounding areas that may not be thought of as urban [15, 16].

Definitions based on geographic extent of built-up areas characterize the physical footprint of the city. An important strength of this approach is that it can be applied systematically across countries and over time to track urban growth longitudinally. In addition it captures the boundaries of urbanized areas in a systematic and data-driven fashion [14, 19, 23, 24]. A key disadvantage is that it may be difficult to link other data such as census data or health data to these units because the boundaries identified do not necessarily correspond to any type of administrative area.

#### SALURBAL Approach to Identifying and Operationalizing Cities

Recognizing the complexity of defining cities and the need to be rigorous but practical in order to capitalize on easily available health data, SALURBAL used an approach that combines various criteria. First, we identified the universe of cities of interest. Second, we operationalized cities and their component units so that various data sources could be linked to them. We used a three-level tiered system to define cities and their subunits. We labeled “cities” as “level 1,” sub-city components as “level 2,” and neighborhoods as “level 3.”

##### First Step: Identifying the Universe of SALURBAL Cities

The project identified “cities” with ≥ 100,000 inhabitants as of 2010 in the 11 SALURBAL countries as the universe of interest (here we use the term “cities” in quotes broadly to refer to units that may be an urban agglomeration or some form of administratively defined cities). The countries currently included in the SALURBAL cities platform are Argentina (AR), Brazil (BR), Chile (CL), Colombia (CO), Costa Rica (CR), El Salvador (SV), Guatemala (GT), Mexico (MX), Nicaragua (NI), Panama (PA), and Peru (PE). A cut-off population size of 100,000 inhabitants was selected because it is a threshold often used to define cities and allows the inclusion of “cities” of varying size [14–16, 20, 25]. Not all “cities” will be included in all analyses as there will likely be important heterogeneity in the data available to answer a given research question, but identifying the universe is critical to provide context for results.

We created a draft list of “cities” with 100,000 inhabitants or more by combining information from two sources: The 2010 Atlas of Urban Expansion (AUE) and a database of census data compiled at http://citypopulation.de (henceforth referred to as CP). The 2010 AUE [14] included 377 “cities” determined to have 100,000 population or more in the 11 SALURBAL countries. Because the AUE defines cities approximately based on their built-up area (analogous to the third definition above), the “cities” include both urban agglomerations (collections of nearby administratively defined areas) and single administratively defined cities. The CP is dedicated to collecting census data from countries worldwide, including lists of cities and other urban settlements. It is regularly updated with local population estimates [26]. Cities are defined based on a country’s administrative definitions such as a municipality or “a populated center, locality, or an urban area within a municipality.” The preferred year of population counts (or projections) was 2010 to match with the AUE population estimates. The CP list included 539 cities with population ≥ 100,000 in 2010 in the 11 SALURBAL countries.

We matched the AUE list of cities to the CP list by city name, country administrative sub-divisions, and country. All AUE-defined “cities” had a match in the CP list, but not all cities in the CP list matched to an AUE “city.” Satellite imagery in Google Earth (Google, Inc., Mountain View, California), NASA Earth Observatory Night Light Maps 2012 (NASA Worldview application, https://worldview.earthdata.nasa.gov/), and population data from both sources were used to assess whether the cities on the CP list that did not match the AUE list were actually already part of a larger AUE urban agglomeration. If an unmatched city was not part of an AUE defined city, it was added to the list. The final result was a consolidated list of “cities” of ≥ 100,000 population that integrated information from both databases.

**Fig. 1:**
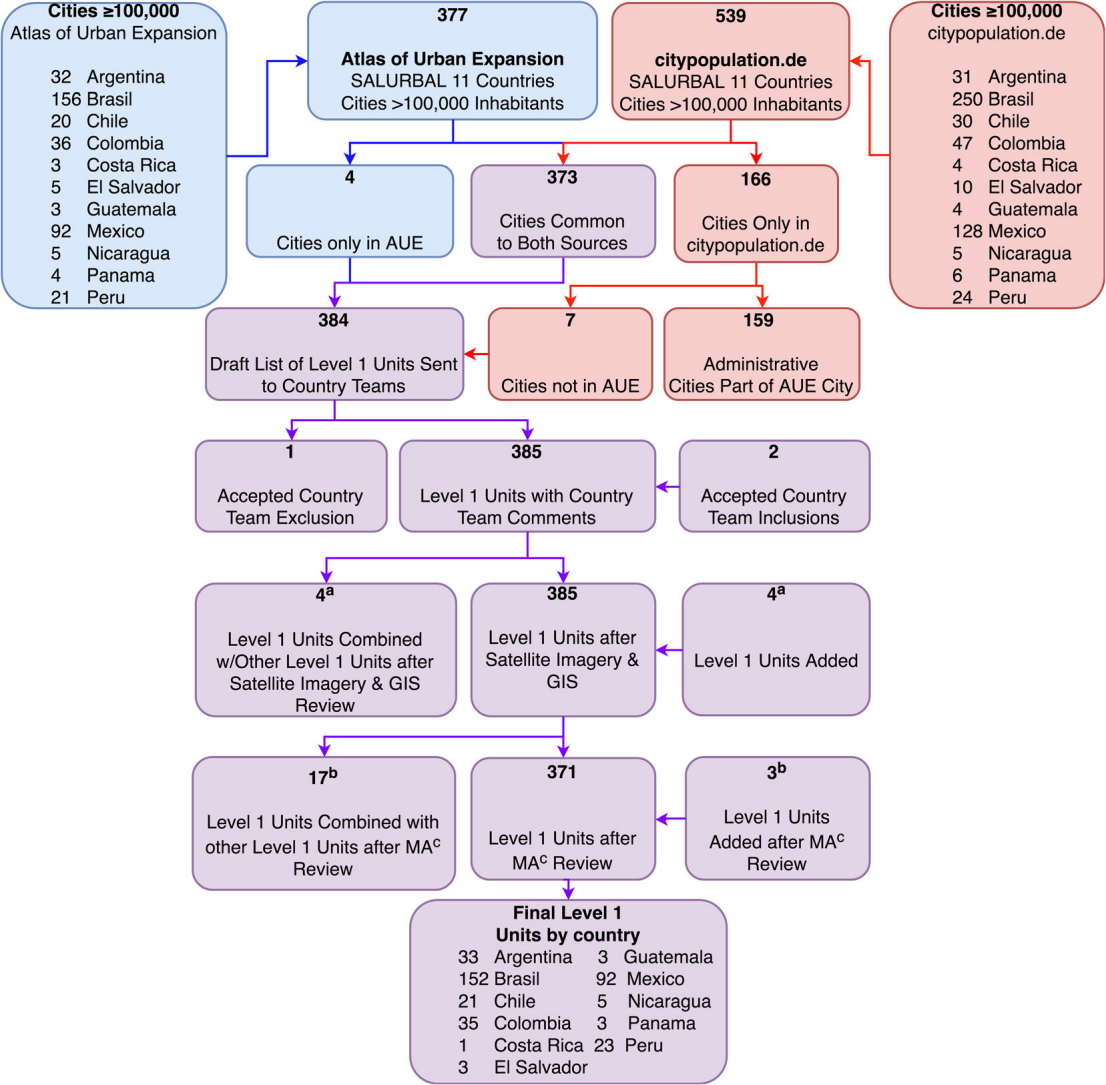
The process used to identity “cities” in 11 SALURBAL countries. Footnotes: (a) During the operationalization of cities as clusters of L2 units (see section on definition of L1Admin), it was observed that some L1 “cities” shared contiguous built-up areas. This resulted in adjacent L1 units being combined with other L1 units (*N* = 4) to create a consolidated “city”. Additionally, some administrative cities with populations of less than 100,000 were observed to share contiguous built-up areas with other nearby administrative cities such that together these units met the population eligibility requirement. This resulted in the addition of a small number (*N* = 4) of L1 units. (b) As a result of comparing the list of cities with what some countries deem as “metropolitan areas,” 3 new L1 units were added and 17 were merged with other L1 units. (c) MA = metropolitan areas

**Fig. 2:**
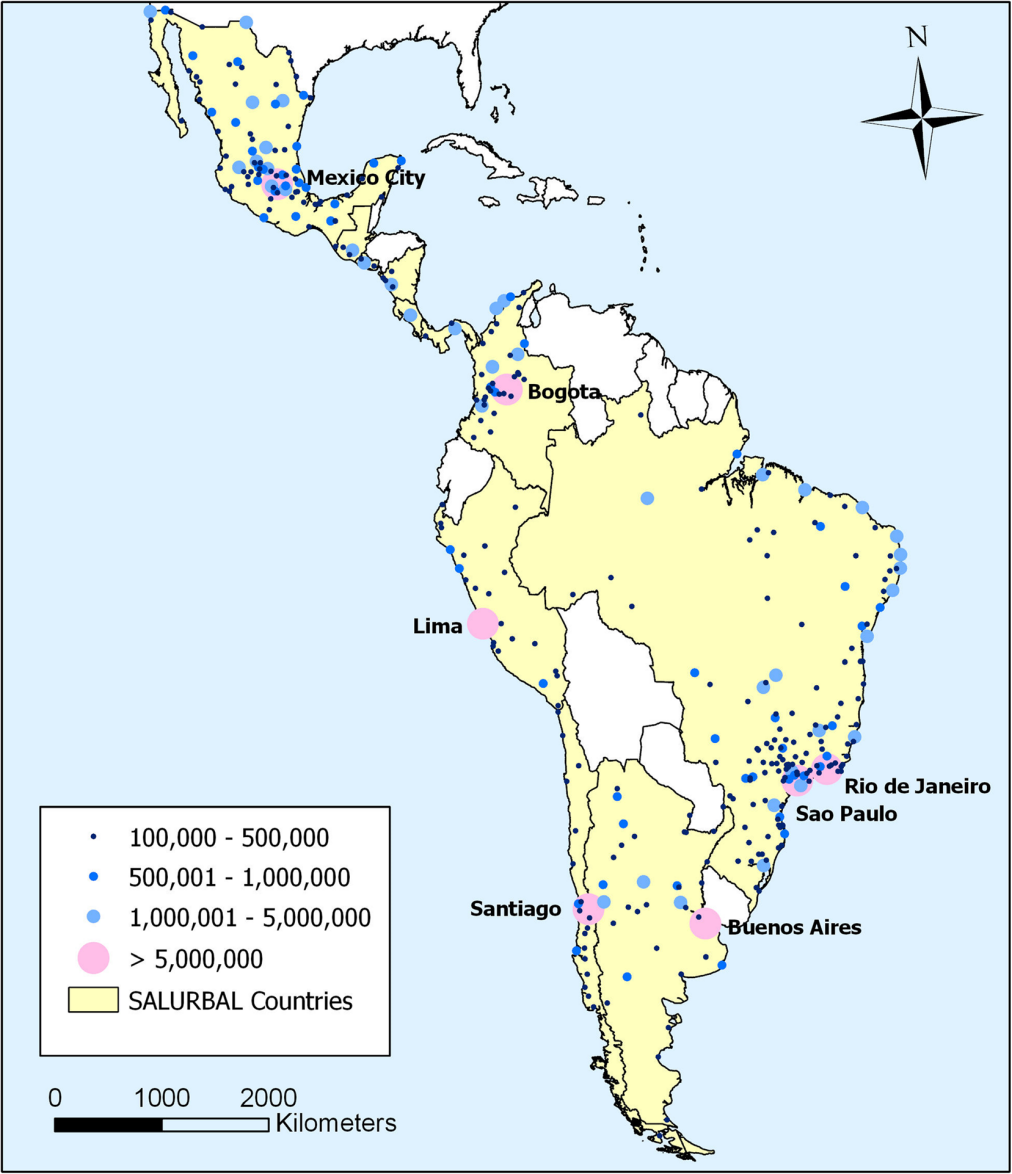
Map of SALURBAL countries and cities

The draft list of “cities” was reviewed by each country team for face validity resulting in a few minor modifications to the list. A few additional modifications to the list were made as a result of the operationalization of these “cities” as clusters of smaller sub-city units (which we describe below further) and as a result of the comparison of this list to country-defined metropolitan areas. The full process used to arrive at the final list of 371 “cities” is summarized in Fig. 1 and shown geographically in Fig. 2.

##### Second Step: Creating Complementary Operational Definitions of “Cities” and Subunits Within Them

SALURBAL created four complementary definitions of “cities” or level 1 units: (1) L1Admin: based on the built-up urban extent approximated through clusters of administratively defined areas; (2) L1Metro: based on country specific definitions of metropolitan areas; (3) L1UrbExt: based on the precise built-up urban extent identified systematically using satellite imagery; and (4) L1Excess: similar to L1UrbExt but including urban extents that spill over to neighboring non-SALURBAL countries, (for example Tijuana, Mexico’s built area spilling into San Diego, USA). In addition to defining “cities,” SALURBAL also defined sub-city units (level 2 or L2) and neighborhoods within cities (level 3 or L3). A summary of the SALURBAL geographic definitions and “levels” is provided in Table 1.

**Table 1:** SALURBAL definitions of cities and their component units at various levels

Level	Definition
Level 1 “city”	
L1Admin (administrative)	“City” defined as a single administrative unit (e.g., municipio) or combination of adjacent administrative units (e.g., several municipios) that are part of the urban extent as determined from satellite imagery. Each L1Admin is defined based on its component level 2 units.
L1Metro (metropolitan areas)	“City” defined following the exact definition that each country provides for metropolitan areas (if available), as a combination of either level 2 units or other units.
L1UrbExt (urban extent)	“City” defined based on systematically identified urban extent based on built area; boundaries may not overlap exactly with administrative units.
L1Excess (urban extent spillover)	“City” defined as in L1UrbExt but also including the urban extent spilling into a neighboring non-SALURBAL country.
Level 2 “sub-city”	Administrative units (e.g., municipios) nested within L1Admin. In some cases, this may be a single unit for each city, and in other cases, it will be multiple units. In some cases, level 2 units may also be nested within L1Metro.
Level 3 “neighborhood”	Smaller units such as census tracts that can be used as proxies for “neighborhoods” within a city. Level 3 units will be nested within level 2 units. They will also be approximately linked to L1UrbExt so that census data can be linked to the L1UrbExt for analyses. In some cases, level 3 units may also be nested within L1Metro.

##### Defining L1 Administrative Units and Their Component Subunits

In order to link city data with health data, it was critical to have a practical definition of “cities” that could be operationalized as clusters of the smallest geographic units for which health data was either publicly available or easily available upon request (i.e., without requiring georeferencing). We therefore identified the “level 2” units (L2) in each country as the geographic administrative units for which health data was easily available and then proceeded to link each “city” on our list to the corresponding L2 units. Some “cities” encompassed only one L2 unit and others included multiple L2 units. In general, L2 units were defined as *comunas*, *municipios*, or similar units depending on the country. The cluster of L2 units that were attached to a given L1 was labeled the L1Admin.

A L2 unit was considered to be part of an L1Admin if it covered at least part of urban extent (initially determined by visual inspection of administrative boundaries and satellite imagery and then refined when the L1UrbExt was defined, see below). We included all L2 units that included any portion of the urban extent, even if they also captured areas outside the urban extent. In many cases, the population of the L2 unit will likely lie mostly within the most urbanized area. Subsequently, sensitivity analyses excluding L2 units that are not fully urban (based on census data) or that are only partly include the urban extent can be conducted. In cases where a L2 unit covered more than one “city,” it was assigned to the “city” with which it shared the largest amount of built-up area.

We identified neighborhoods or L3 units based on census hierarchies within each country. We looked for units that were comparable in size and that were nested within L2 units. L3 units facilitate examination of within-city variability when georeferenced health data are available and constitute building blocks for larger units (L2 units and L1UrbExt units) thus allowing linkage of these larger units to census and other data. In most countries, these units reflect the basic small-area census division for urban areas or for the entire country and were generally defined to facilitate census data collection. In some cases, the administrative units defined as L3 units did not cover the full country and were only available for country-defined “urban areas” (which may not coincide will SALURBAL L1Admin or L1UrbExt). In these cases, SALURBAL developed a strategy for creating SALURBAL defined L3 proxies in areas that were not covered. For details see Appendix Table 8. A summary of the definitions of L2 and L3 units for each country is provided in Table 2. A summary of the numbers of units at each level and their population sizes by country is provided in Table 3.

**Table 2:** SALURBAL cities and definitions of Level 2 and 3 units by country

Country	Cities	Level 2 unit	Level 3 unit^b^
Argentina	33	Departamento/Partido/Comuna^a^	Radio Censal
Brazil	152	Municipios	Setor Censitário
Chile	21	Comuna	Zona Censal
Colombia	35	Municipio	Sector Urbano
Costa Rica	1	Canton	Unidad Geoestadistica Basica
El Salvador	3	Municipio	Sector Censal
Guatemala	3	Municipio	Sector Censal
Mexico	92	Area Geoestadistica Municipal	Area Geoestadistica Basica
Nicaragua	5	Municipio	Sector Censal
Panama	3	Corregimiento	Barrio
Peru	23	Distrito	Zona Censal

^a^Comunas in the Ciudad de Buenos Aires, Partido in the Provincia de Buenos Aires, Departamentos elsewhere

^b^As defined for country-designated urban areas

**Table 3:** Descriptive statistics and population sizes of L1Admin, L2, and L3 units by country. Population for L1Admin and L2 are from 2010 census projections from each country. L3 population sizes are from most recent census data available

	L1Admin			L2				L3			
	*N*	Population (in 1000s)		Total *N*^a^	Units per L1Admin		Population (in 1000s) Median (5th–95th percentile)	Total *N*^a^	Units per L2		Population Median (5th–95th percentile)^g^
		Median (5th–95th percentile)	Max		Median (5th–95th percentile)	Max			Median (5th–95th percentile)	Max	
AR	33	304.2 (123.0–1466.5)	14,791.1	110	1 (1–6)	51	188.8 (28.8–605.7)	29,792	218 (34–606)	1493	883 (250–1692)
BR	152	231.4 (114.5–3070.4)	19,987.8	422	1 (1–9)	31	124.2 (15.6–798.2)	164,107	183 (23–1074)	18,953	646 (79–1251)
CL	21	215.3 (126.8–994.6)	6213.8	81	1 (1–10)	36	137.9 (27.6–319.2)	3,918^b^	39 (14–114)	172	2200 (0, 7787)
CO	35	360.3 (119.9–2822.2)	8546.8	84	1 (1–6)	15	115.0 (12.8–895.6)	4,679^c^	22.5 (2–170)	643	^h^
CR	1	2367.0 (2367.0-2367.0)	2367.0	29	29 (29–29)	29	57.4 (22.7–251.6)	^f^			
SV	3	261.9 (241.2–1704.8)	1870.8	22	1 (1–18)	20	79.5 (9.3–267.3)	944	28.5 (4–108)	137	2361 (1425-3031)
GT	3	242.0 (150.3–2633.0)	2898.7	20	5 (1–13)	14	94.1 (22.8–516.4)	4025	86 (21–688)	1485	677 (312–1106)
MX	92	351.7 (134.9–1855.9)	20,014.5	406	2 (−15)	76	67.6 (7.1–774.5)	32,921^d^	32 (4–319)	638	1749 (6–5636)
NI	5	174.1 (117.6–936.0)	1120.4	11	1 (1–5)	6	76.8 (20.3–555.8)	^f^			
PA	3	212.0 (209.4–1591.8)	1745.1	82	18 (12–50)	53	20.1 (2.2–66.2)	1,800^e^	18 (1–53)	147	116 (3–1150)
PE	23	281.5 (127.7–876.8)	9177.7	169	5 (2–18)	51	55.9 (4.9–340.0)	^f^			

^a^Total *N* refers to the number of units across all SALURBAL cities

^b^Includes 385 proxy L3 units created by SALURBAL, median units per L2 = 3, max units per L2 = 17

^c^Includes 290 proxy L3 units created by SALURBAL, median units per L2 = 2, max units per L2 = 31

^d^Includes 388 proxy L3 units created for SALURBAL, median units per L2 = 1, max units per L2 = 1

^e^Includes 74 proxy L3 units created for SALURBAL, median units per L2 = 1, max units per L2 = 1

^f^Cartography and population for L3 units pending

^g^Population for L3 are from the following census years by country: AR, BR, MX, and PA are from 2010; CL and GT are from 2002; SV is from 2007

^h^Population for L3 from Colombia 2007 census is pending

##### Defining “Metropolitan Areas” or L1Metro

The second definition of Level 1 “cities,” L1Metro, was based on each country’s official definition of metropolitan areas (or similar areas). The definitions of L1Metro differed by country and are summarized in Appendix Table 9. L1Metro units may include multiple L1Admin units in their entirety or partially. In all countries except Argentina and Peru, L1Metro units are aggregates of L2 units. In Argentina, each L1Metro is composed of *localidades* and in Peru each L1Metro unit is composed of *Centros Poblados*. These units in both countries can be linked to L3 units.

##### Defining L1UrbExt and Its Spillover Extension L1Excess

While a qualitative assessment of the visual urban extent was used to help identify the L2 units linked to each L1Admin, a more refined, systematic, and quantitative approach was needed to properly define the urban extent of each L1 unit*.* This process used the Global Urban Footprint (GUF) Dataset [28, 29] and followed procedures similar to those used by the Atlas of Urban Expansion to define urban extents with some modifications. The GUF is a worldwide mapping product derived using TerraSAR-X and TanDEM-X images, with a spatial resolution of 0.4 arcsec (~ 12 m), which classified pixels as built-up and non-built-up [28]. This classification was achieved by highlighting areas of images characterized by highly diverse and heterogeneous backscattering, then using an automated classifier, and followed by semi-automatic post processing. TerraSAR and TanDEM are two satellites designed to acquire high-resolution and good quality radar images covering the entire earth that are used for a wide range of applications, such as topographic mapping, land cover, and land use change detection [28–30]. In the process of defining urban extent, the pixels were identified as urban, suburban and rural according to the share of built-up pixels within a 1-km^2^ area. Urban clusters were generated by merging the urban, suburban and urbanized open space. A hierarchical agglomerative process was used to join the urban clusters nearby following an inclusion rule. The largest urban cluster in each L1Admin was defined as L1UrbExt.

The L1UrbExt analysis identified four potential cases that required further consideration, and if appropriate, modification of L1Admin definitions. First was when the L1UrbExt extended beyond the geographic boundaries of the L1Admin (as first defined using visual inspection of satellite imagery) and therefore the L1Admin needed to be modified by adding a L2 unit (3 cases). Second, when L1UrbExt extended beyond the geographic boundary of the L1Admin by less than 20% of the L1Admin area, in which case we ignored the extra area (3 cases). Third, when the L1UrbExt spills into another L1Admin, in which a case by case analysis identified that separate L1UrbExts were appropriate (2 cases) and no modifications to the L1Admins were made.

Fourth, when the L1UrbExt spilled into a neighboring non-SALURBAL country (10 cases, spilling into Paraguay, Uruguay, the USA, and Venezuela), we created the level 1 excess (L1Excess) to include the non-spillover plus the spillover area into the neighboring country. This was done because even though health data outside of SALURBAL countries would not be linked to the L1Admin, some measures of the L1UrbExt (such as air pollution) might be relevant to health on the other side of the border.

### Linking Health and Environmental Data at Various Geographic Levels

**Fig. 3:**
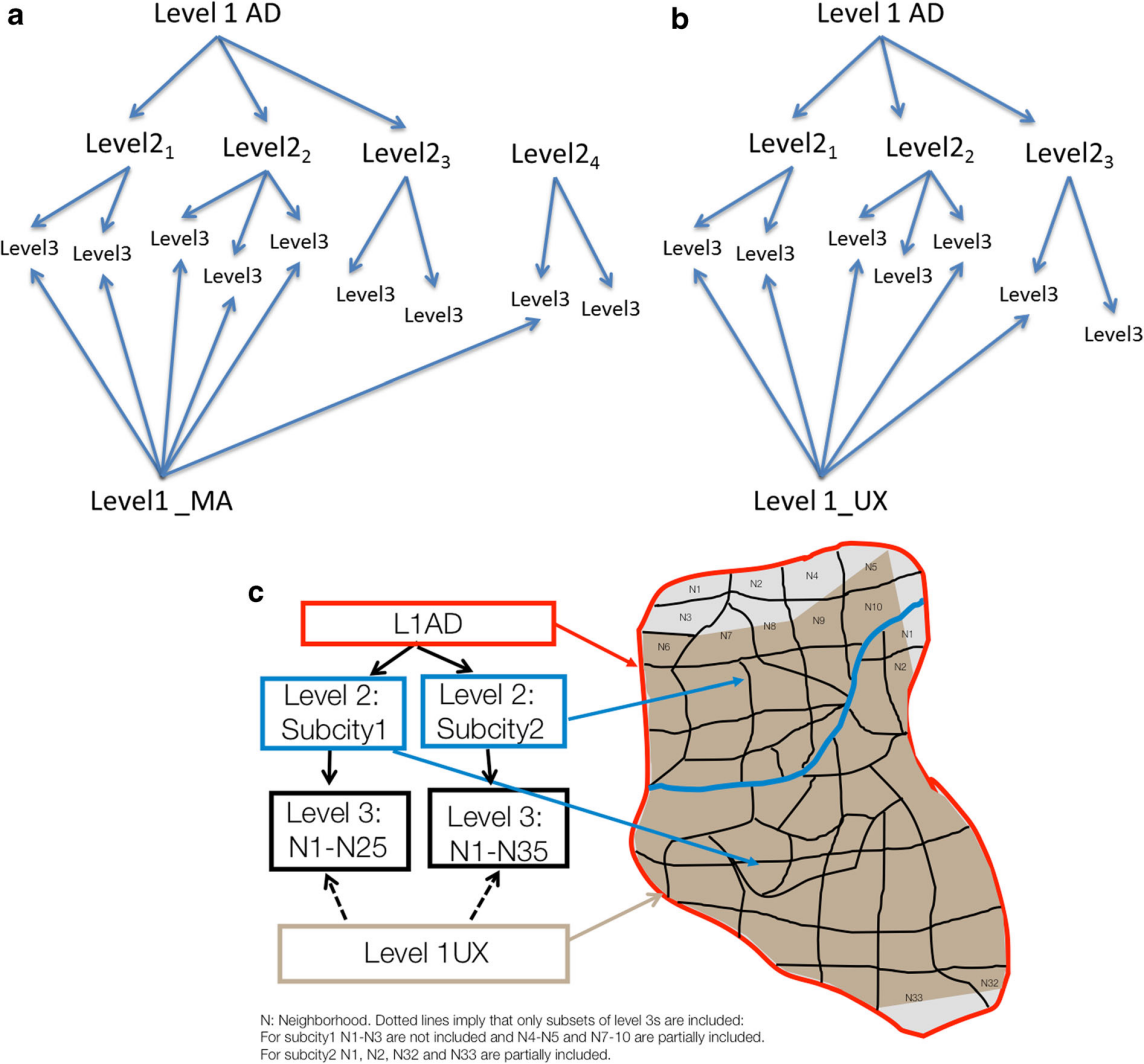
**a** Links between L1Admin, L2, L3, and L1Metro. The L1Metro may or may not overlap with the level 2 units that compose the L1Admin and may or may not include L2 units outside of the L1Admin. Depending on the country, the L1Metro may include all L3 units within L2’s or only selected L3 units within them. **b** Links between L1Admin, level 2, level 3, and L1UrbExt. The L1UrbExt may include subsets of L3 units within the L1Admin. In a small number of cases a variant of the L1UrbExt that extends outside the boundaries of the country (and the L1Admin) was created and called L1Excess. **c** Spatial representation of links between L1Admin, L2, L3, and L1UrbExt. L1Metro is not shown but may include L2s or L3s beyond the L1Admin or may encompass only part of the L1Admin.

A summary of the geographic hierarchies and possible linkages using the SALURBAL geographic levels is provided in Fig. 3. The L1Admin, level 2, and level 3 hierarchy is straightforward as units are nested within each other (Fig. 3). In many cases, L1Metros are also clusters of L2 units, although they are sometimes larger and may encompass a different set of L2s than the L1Admins (Fig. 3). In countries where L1Metros are not defined using L2s (Argentina and Peru), they can be defined using L3s (Fig. 3a). L1UrbExts will be approximately linked to L3s (Fig. 3b). L3 units will be considered part of a L1UrbExt if they contain any portion of the area of the L1UrbExt. If necessary, weights may be used to attribute L3 data to the L1UrbExt in cases where the L3 is only partly covered by the L1UrbExt. A spatial representation of these linkages is shown in Fig. 3c. These data structures facilitate linkages of health and environmental data at various levels. They also allow for differences across data and countries in the spatial resolutions available. SALURBAL is in the process of georeferencing mortality and survey data to L3 whenever possible, thus allowing for analyses at finer spatial resolution. In the meantime, analyses based on L1Admins or L2s can proceed as aggregate data for these units is more readily available.

The data structure proposed can be expanded to include time-varying health and environmental data linked to various geographic units. This is easily accomplished by adding calendar year indicators to spatial IDs. A challenge will be harmonizing units in cases where spatial definitions of administratively defined geographic units (such as L2 units, L3 units, or L1Metros) have changed over time. Definitions of L1UrbExts are designed to change over time in order to capture longitudinal changes in urban extent. If feasible, SALURBAL may explore approaches to harmonize geographic boundaries of selected units over time, as has been done in the USA [31, 32].

## Obtaining and Harmonizing Health Data

### Mortality Data

We obtained individual-level mortality records at L2 from each country (except Nicaragua) for as many years as possible. These records included at least age, sex, location of residence, and cause of death. Most countries had data on education of the decedent. We harmonized all variables to guarantee comparability. Sex was categorized as male, female, or missing. Age was operationalized in single-year intervals whenever possible (all countries except Colombia). Education was harmonized using the IPUMS international recode [33]. Causes of death were coded using either ICD9 or ICD10 codes (depending on the year) and grouped in categories using the World Health Organization Global Health Estimates (GHE) classification [34].

Three potential issues challenge the quality of mortality data, and we evaluated and addressed each one as follows. First, some mortality records have missing information on the variables of interest (age, sex, cause of death, location of residence, and education). To evaluate this issue, we computed missing data proportions for each variable by country and year (see Appendix Table 10). To impute these missing values, we used conditional probabilistic imputation by sex and cause of death (for age), by age and cause of death (for sex), and by age and sex (for cause of death), all stratified by country and year. For example, records with missing age or sex were imputed to a 5-year age category or to male or female probabilistically, based on the observed distributions of each variable in their corresponding sex and cause of death (for age) or age and cause of death (for sex). Records with missing cause of death were imputed to either ill-defined diseases or injuries of ill-defined intent (see below), probabilistically by age and sex. Mortality records with missing location of residence at L2 were dropped, as these would not be linkable to a SALURBAL area.

Second, some mortality records had a cause of death coded as an ill-defined disease (e.g., R chapter of the ICD10 classification) or as an injury of ill-defined intent (e.g., codes Y10–Y34 and Y872 in the ICD10 classification). We evaluated this issue by computing the proportion of all deaths that were coded as ill-defined diseases or injuries of ill-defined intent (see Appendix Table 10). Given that these ill-defined deaths make it challenging to estimate the public health burden of diseases and injuries, we redistributed them to other GHE categories proportionally by age, sex, country, and year. This approach is similar to that used by the GHE study [34].

Third, not all deaths that occur in a country are registered in a vital registrations system. The phenomenon of lack of complete coverage or undercounting biases down the estimates of mortality. We evaluated this issue by obtaining estimates of undercounting from the United Nations Development Program (see Appendix Table 11). These estimates apply to the entire country, so we obtained more detailed estimates wherever possible. This is especially important in countries with wide geographic variability and high rates of undercounting such as Peru and Colombia, where (a) a national estimate of undercounting my underestimate or overestimate the lack of coverage and (b) this differentiation may be meaningful (as the overall rates are high). In countries where this distinction was less relevant, we applied a blanket correction for the entire country. Appendix Table 11 details the specific corrections we applied to each country, whether they are L2 specific (or at a higher level) and whether they are age or sex specific. Overall, we applied these correction factors by using them to estimate the number of missing deaths (for the entire country or each L2, for all age groups or a specific age group, and for both sexes or each specific gender, see Appendix Table 11). Once we estimated the number of missing deaths, we sampled this number with replacement (hot deck imputation) from the observed deaths following similar procedures as the GHE.

The final product was a collection of datasets with information on each individual mortality record, including year, country, location of residence (at L2), age (in single or 5-year groups), sex, education (if available), and cause of death (3 variables: ICD-10 code, GHE classification, and GHE classification with redistributed ill-defined diseases and injuries of ill-defined intent). Moreover, we created an aggregated dataset, summing the number of deaths in each year, L2, 5-year age category, sex, education (if available), and cause of death using the GHE classification (with and without applying the redistribution of ill-defined diseases and injuries of ill-defined intent). These aggregated datasets contained both the number of deaths corrected for lack of complete coverage and the uncorrected number of observed deaths.

### Population Data

In order to use mortality records to estimate mortality rates, we had to obtain estimates of the population counts by year, location of residence (L2), age, and sex. These population projections were obtained from the census bureaus of each country. In most countries, estimates by age and sex were available at L2. In some cases (Peru and El Salvador), estimates by age and sex were only available at higher administrative levels instead of L2, while data for L2 was available by either age or sex. In these cases, we estimated L2 population counts by age and sex by redistributing the counts by age or sex to the proportions observed at higher levels. More details are available in Appendix Table 12.

### Survey Data

SALURBAL plans to compile health surveys and any available cohort studies in order to develop harmonized measures of health behaviors and other risk factors. Our initial focus has been on national health surveys with a focus on non-communicable disease risk factors. The design and sampling approaches differ somewhat across countries, but all allow linkage to SALURBAL L2 units (and may in the future also allow linkages to L3 units). Some surveys are based only on self-report information, but others include objective measurements such as height, weight and blood pressure [35]. A data harmonization effort was launched to create comparable measures of selected domains. The design of the surveys implies that their geographic level or representativeness may differ (Appendix Table 13). This will be taken into consideration if prevalence estimates for specific cities are generated. In addition, we will use statistical approaches that can be leveraged to derive small area estimates even when the survey was not specifically designed for that purpose [36–39]. For the most part, however, survey data will be used in multilevel analyses to estimate associations of city or neighborhood-level factors with individual-level outcomes. Sampling design and weights will be taken into consideration, if appropriate, as has been done in prior work [40–43]. Appendix Table 13 summarizes methodological and geographic characteristics of surveys selected for initial harmonization.

SALURBAL developed a process for harmonization of priority domains that included the following: (1) identifying and collating questions and responses by domain, with attention to skip patterns and respondent universe; (2) reviewing surveys conducted by others such as the Centers for Disease Control and Prevention or the World Health Organization for standard variable definitions as well as harmonization approaches proposed by other projects [33, 44, 45]; (3) proposing harmonized variable definitions and response categories with attention to differences in wording across countries; and (4) applying the harmonization and revising the protocol as needed, based on descriptive statistics of initial harmonized variables. In some cases, multiple versions of a variable were created due to country differences that did not allow a single harmonized variable. The harmonized data will be linked to L2 and L3 whenever possible. In addition SALURBAL is exploring other methods to combine heterogeneous data across countries using approaches, such as differential item functioning [46], meta-analysis approaches [47, 48], and fused LASSO models or other machine learning approaches [49]. Priority domains of interest and variable definitions are shown in Table 4. Other domains will be harmonized as the study advances.

**Table 4:** SALURBAL health survey domains and selected measures.

Domain	Variables	Definitions	Source^a^
Demographics	Age	Age in years	N/A
	Sex	Male or female	
	Education	Education level as less than primary, primary completed, secondary completed, or more than secondary completed	IPUMS-I [33, 44]
Diabetes	Diabetes	Presence of diabetes diagnosis by a health care provider among all adults (excluding diagnoses during pregnancy)	CDC [50] WHO [51, 52]
	Gestational diabetes	Presence of gestational diabetes diagnosis among all adult female respondents with a history of pregnancy	
	Diabetes treatment	Any pharmacological treatment among those with diabetes	
Hypertension	Hypertension	Presence of hypertension diagnosis by a health care provider among all adults (excluding a diagnosis during pregnancy)	CDC [53] WHO and NCD RisC [54] WHL [55]
	Gestational hypertension	Presence of gestational hypertension diagnosis among all adult female respondents with a history of pregnancy	
	Hypertension treatment	Any pharmacological treatment among those with hypertension	
	Systolic blood pressure (SBP)	Average of 2–4 SBP measured by survey interviewer	
	Diastolic blood pressure (DBP)	Average of 2–4 DBP measured by survey interviewer	
Health status	General health status	Respondent’s self-rated health categorized as very poor to very good or excellent	OECD [56] CDC-BRFSS [57]
Tobacco use	Cigarette smoking status	Cigarette smoking status as current, former, or never smoker among adults	CDC [58] GTSS [59]
Alcohol use	Binge drinking	Varied by country: defined as 3 or 4 or 5 alcoholic drinks for women and 4 or 5 alcoholic drinks for men in the past 30 days on one occasion	CDC [60] WHO [61]
	Current drinking (30 days)	Any consumption of alcoholic beverages in the past 30 days	
	Current drinking (12 months)	Any consumption of alcoholic beverages in the past 12 months	
Anthropometrics	Height (measured)	Measured	WHO [62]
	Weight (measured)	Measured	
	Height (self-reported)	Reported by respondent	
	Weight (self-reported)	Reported by respondent	
	Body mass index (BMI based self-reported or measured height and weight)	Reported by respondent or measured	
Physical activity	Global physical activity	Total minutes of self-reported physical activity in the past week	IPAQ [63] GPAQ [64]
	Transportation physical activity	Total minutes of self-reported transportation-related physical activity in the past week	
	Leisure physical activity	Total minutes of self-reported leisure physical activity in the past week	
	Total walking	Total minutes of self-reported walking in the past week	
Nutrition	Fruit consumption frequency	Number of days per week in the last week	WHO [65] IARC [66] CDC [67]
	Vegetable consumption frequency	Number of days per week in the last week	
	Soda consumption	Number of days per week in the last week	
	Dessert foods consumption	Number of days per week in the last week	

*IPUMS-I* Integrated Public Use Microdata Series, International, *CDC* Centers for Disease Control and Prevention, *WHO* World Health Organization, *GTSS* Global Tobacco Surveillance System, *NCD RisC* Non-Communicable Disease Risk Factor Collaboration, *OECD* Organisation for Economic Co-operation and Development, *WHL* World Health League, *BRFSS* Behavioral Risk Factor Surveillance System, *IARC* International Agency for Research on Cancer

^a^Data source used to inform harmonized definition

## Characterizing Urban Social and Physical Environments

Several key social and physical environment domains were identified as potentially relevant to health and health inequalities in cities by the SALURBAL team. The domains as well as selected indicators for these domains and the data sources that are being used to estimate them are summarized in Tables 5 and 6. Indicators may be defined for L3, L2 or L1Admin, L1Metro, and L1UrbExt based on the construct and data availability.

**Table 5:** Social environment domains and indicators

Domain	Indicator	Definition	Level	Data source(s)
Economic				
Poverty, income, and inequality	Poverty	Proportion of population living below the nationally defined income-based poverty level	L1–L3	Census or national household surveys
	Income-based Gini Index	A measure of inequality in the distribution of income	L1	Census or national household surveys
Employment	Unemployment	Proportion of persons 15 years or older in the labor force who are not working but seeking employment	L1–L3	Census or national household surveys
	Labor force participation	Proportion of persons 15 years or older who are working or seeking employment	L1–L3	Census or national household surveys
Social				
Education	15–17 years old in school	Proportion of 15–17 year-olds enrolled in school	L1–L3	Census
	Adults with completed secondary education or more	Proportion of people 25 years and older with completed secondary education or higher	L1–L3	Census
	Education-based Gini Index	A measure of inequality in the distribution of education	L1	Census
Gender empowerment	Female labor force participation	Proportion women 15 years or older who are working or seeking employment	L1–L3	Census or National household surveys
	Female government leadership	Proportion of city leadership (e.g., city council members) who are female	L1	National government sources
Violence and disorder	Violent deaths	Age-standardized homicide rate per 100,000 population of homicides	L1–L2	Mortality
	Crime/safety	Proportion of individuals reporting being a victim of a crime in the past 12 months Safety perception score	L1–L2	Selected national surveys, CAF Survey [68]
	Social disorder	Social disorder/incivilities scale	L1–L2	CAF Survey
Social cohesion and social capital	Election participation	Proportion of eligible individuals voting in the last presidential election	L1–L2	CAF Survey
	Community organization membership	Proportion of individuals who are part of a community or neighborhood organization.	L1–L2	CAF Survey
	Neighborhood connectedness	Neighborhood connectivity scale/social support scale	L1–L2	CAF Survey
	Discrimination	Proportion of individuals reporting discrimination	L1–L2	CAF Survey
Housing				
	Water connection	Proportion of households without piped water	L1–L3	Census
	Sewage connection	Proportion of households lacking a connection to the municipal sewer system or a septic tank	L1–L3	Census
	Overcrowding	Proportion of households with 3 people per room or more	L1–L3	Census
	Housing materials	Proportion of households with non-durable wall materials	L1–L3	Census
Governmental, institutional, and organizational				
	Governance	Presence of participatory budgeting	L1	Selected national sources
		Property taxes: total revenue and as % of GDP and total tax revenue	L1/L2	Lincoln Land Institute
	Social services and health care	Percent of population with health insurance	L1	Selected country surveys
		Percent of children with age-appropriate vaccine coverage	L1	Selected country surveys
		Percent of households in poverty receiving public assistance	L1	Selected country surveys

Additional indicators under exploration/development include city GDP, presence of various land/climate/energy/disaster/transit policies/plans, % housing in informal settlements, minimum wage, cell phone subscription rates, and health care service/provider availability

**Table 6:** Physical and built environment domains and indicators

Domain	Definition	Indicators	Level	Data source
Urban form and population metrics				
Population	Measure of the number of people living per unit of an area or within a geographic boundary	Total population, population density, Gini coefficient of the population distributions	L1–L2	Census or population projections^a^
Population distribution	Measure of concentration population within geographic boundary	Gini coefficient of population distribution	L2–L3	WorldPop^b^ [69]
Neighborhood centrality	Measure of the distance to the city center	Neighborhood centrality	L2–L3	Local sources
Urban landscape metrics				
Area	Measure of the urbanized area inside a geographic boundary	Total urban area, percentage of urban area, coefficient of variation of urban patch^b^ area, area-weighted mean urban patch area, mean urban patch area, effective mesh size	L1–L3	Global Urban Footprint (GUF) Dataset derived by TerraSAR-X and TanDEM-X images [28, 29]
Shape	Measure of compactness and complexity	Area-weighted mean shape index		
Fragmentation	Measure of fragmentation of urban expansion. It is the relative share of open space in the urban landscape	Number of patches, patch density, mean patch size, effective mesh size		
Isolation	Measure of the tendency for patches to be relatively clustered or isolated in space. It is the mean distance to the nearest urban patch within the geographic boundary	Area-weighted mean euclidean nearest neighbor distance		
Edge	Measure of fragmentation and shape complexity. It is the boundary between urban and non-urban patches	Edge density, area-weighted edge density		
Aggregation	Measure of the tendency of clumping of urban patches	Aggregation index		
Street design and connectivity metrics				
Street density	Measure of street network density	Street density, large road density	L1–L3	OpenStreetMap and OSMNx [70]
Intersection density	Measure of the amount of intersections within the street network	Intersection density, intersection density 3-way, intersection density 4-way, streets per node average, streets per node standard deviation		
Street network length and structure	Measure of street network structure	Street length average, circuity average		
Transportation metrics				
Bus rapid transit	Bus-based transit system that includes dedicated lanes, traffic signal priority, off-board fare collection, elevated platforms, and enhanced stations	Presence of BRT, BRT length, BRT daily users, BRT price per ride, BRT supply length, BRT demand, BRT payment capacity	L1–L3	BRTData, OpenStreetMap, minimum wage of Latin America and local sources
Subway, light rail, and/or elevated train (SLRET) transport systems	Mass rapid transit, including heavy rail, metro or subway	Presence of SLRET, SLRET length, SLRET daily users, SLRET price per ride, SLRET supply length, SLRET demand, SLRET payment capacity		OpenStreetMap and local sources
Aerial Tram transport system	Transport lift systems integrated into the city’s public transport network that provide mobility options for those living in hillside neighborhoods	Presence of aerial tram, aerial tram length		OpenStreetMap and local sources
Bicycle facilities	Public infrastructure for exclusive or shared use of bicycles	Total length of bike lanes, bike lane km per population, presence of Open Streets program and length of Open Streets programs		OpenStreetMap, CAF data, and local sources
Urban travel delay index	Measure of congestion	Measures the increase in travel times due to congestion in the street network	L2	OpenStreetMap and Google Maps Distance Matrix API
Gasoline price	Adjusted gasoline price	Price per gallon adjusted by minimum wage	L1	Local sources
Air pollution and green space metrics				
Parks and green space	Measures of parks or green space availability	Parks area, parks density	L1–L3	Local sources
PM10, NOx, SO4, O3	Annual mean value by existing monitoring station	Annual average in μg/m^3^	L1–L3	Local sources^d^
PM2.5	Annual mean value from satellite measurements	Annual average in μg/m^3^	L1–L3	Dalhousie University [71–73]
Food environment				
Density of chain supermarkets	Large food stores with availability of processed foods, frozen foods and fresh produce	Number of supermarkets /area	L1–L3	Online searches of chain company websites
Density of chain convenience stores	Stores with long opening hours and high availability of ultra-processed foods	Number of convenience stores/area	L1–L3	Online searches of chain company websites

^a^Population for the urban extent (L1UrbExt) was estimated based on the ratio of built area in the urban extent to the total built area in each L2 unit. Estimated populations for each built-up L2 unit were then aggregated up to the L1UrbEx

^b^Although we found that WorldPop’s downscaled data performed poorly in a few cases, we assumed that WorldPop’s relative concentration of population within a given unit would be representative of the actual population concentration. A measure of disagreement between WorldPop and Census data is included in our data to describe uncertainty in the Gini coefficient resulting from WorldPop population data

^c^A patch is defined as a homogeneous region of a specific land cover type that differs from its surrounding

^d^These air pollution measures are from air quality monitors maintained by local governments

## A Typology of Multilevel Urban Health Questions

The data structure created by SALURBAL can be flexibly used to answer a number of different types of research questions relevant to understanding the drivers of urban health in cities and the policies that may be most effective in improving population health and reducing health inequities. By capitalizing on heterogeneity across cities and within cities, we can identify important city-level and neighborhood-level drivers of variability in health and in health inequities thus obtaining clues on causes of population health and health inequities.

The types of questions that can be explored with the data platform we developed include, for example (1) questions about factors associated with between-city differences in health; (2) questions about factors associated with within-city (neighborhood) differences in health; (3) questions about the impact of city context on inequities in health; and (4) longitudinal questions about factors associated with changes over time at the city or neighborhood level. By exploring these questions, we will obtain evidence important to identifying what strategies can be used by cities to promote health and health equity. A simplified typology of selected questions is shown in Table 7. Many additional possibilities will be possible.

**Table 7:** A typology of selected urban health questions that can be investigated with the SALURBAL data platform

Question	Analytical approach and unit of analysis	Example
Between-city differences		
How much do summary health indicators vary across cities (within and between countries) and what factors are associated with this variability?	Multilevel analysis of city-level outcomes nested within countries (including variables at L1 and at the country level)	Does life expectancy vary across cities? Are these differences associated with city size and recent growth?
How much does individual-level health vary across cities and what factors are related to this variability?	Multilevel analysis of individual-level survey outcomes nested within cities and countries (including variables at the individual level, at L1, and at the country level)	Does the probability of having diabetes vary across cities? How do individual-level factors, city, and country characteristics contribute to these differences?
Within-city differences		
Description of small area variations in summary health within large cities and factors associated with this variability	Small area estimation methods for mortality or survey estimates and their association with neighborhood (L3) characteristics	How much does life expectancy vary within a city? Is this related to area-level poverty?
How much does individual-level health vary across neighborhoods within cities and what factors are related to this variability?	Multilevel analysis of individual-level survey outcomes nested within neighborhoods (L3) and cities (L2 or L1), including variables at the individual-level, and at L3, L2, and L1 as appropriate	How do neighborhood features of the built environment associate with differences in physical activity levels? Do city-level factors (such as street connectivity) modify these associations?
Impact of city context on inequities	Multilevel analysis of city-level outcomes stratified by education nested within countries (including variables at L1 and at the country level) or multilevel models for aggregate data Multilevel analysis of survey respondents nested within cities, including variables at the individual level, city level, and country level	Do mortality differences by education vary across cities? What city-level factors are associated with greater or smaller inequities? Do educational differences in diabetes prevalence vary across cities? Are city-level factors associated with smaller or larger inequities?
Changes over time		
What longitudinal trends in summary health indicators are observed and to what extent do city or country characteristics modify these trends?	Longitudinal analyses of summary city-level health outcomes and their association with time invariant and time-varying city and country characteristics	How has life expectancy changed over time in cities? Are city growth and air pollution levels related to these trends?
Are changes over time in city or neighborhood characteristics related to changes in individual-level health outcomes?	Longitudinal analyses of individual-level survey responses nested within neighborhoods and cities and their relation to L1, L2, or L3 time-varying characteristics	Do changes in a city’s urban landscape and in neighborhood crime levels affect changes in BMI?

## Challenges

### Data Availability, Heterogeneity, and Quality

>Finding and obtaining the data necessary to answer important questions about environments and health in cities remains an important challenge. For example, mortality data at L2 have been generally easy to obtain, but health survey data have been more complicated to access, even for larger geographic areas, like L2 units. Social and physical environment data have to be compiled from multiple heterogeneous data sources with differences across countries in what information is available. Although many countries have rich health surveys, details on the wording of the questions and the skip patterns used can make harmonization difficult. Data quality also varies both within countries and between countries. The team has devised strategies to address quality issues whenever possible via evidence-based corrections (as described for the mortality data) or through sensitivity analyses.

### Spatial Resolution

The informativeness of health data is maximized if the data can be georeferenced. Currently, most SALURBAL data are available at L1Admin and L2, though each country team is advancing efforts to geocode mortality, live births, and health data to at least L3. The challenges of georeferencing have included coming to agreement with appropriate government institutions, selecting a method for georeferencing and a high-quality source of geocoding while maintaining confidentiality, and obtaining the appropriate geodatabases of the geographic boundaries of the L3 or smaller units.

### Longitudinal Data

A goal of the SALURBAL project is to be able to measure changes in the physical and social environment over time and their effect on health outcomes. Some countries will have more data going further back in time than others. While some data may be available going back 20 or 30 years or more, the quality of older data may not be suitable for the project or may not be available at the city or smaller spatial resolution levels; thus, some longitudinal analyses may not include all countries or all cities. Accommodating differences in spatial definitions of L1Admins and other units over time will also present important challenges.

## Conclusion

The creation of this unique data platform presents enormous opportunities for research, capacity building, and policy impact and positions SALURBAL as an example of an integrated comprehensive approach to characterizing and studying the drivers of urban health in low and middle income countries. The flexible, multilevel data structure allows for heterogeneity in space and time at various scales and can accommodate data available with varying degrees of space and time resolution. Various geographic definitions of cities allow for flexibility in analyses depending on research questions and data availability. Additional health data spanning multiple types of health outcomes across multiple ages can be easily incorporated. The data resource will allow a number of analyses to identify factors related to health, health equity, and environmental sustainability of cities. In addition, it is a rich resource for capacity building in the region. The use and presentation of these data (with all its limitations) will necessarily spur improvements to the regional data systems. In addition, continuous updates to the data resources, including addition of other health outcomes across the lifecourse and the incorporation of data on the timing and characteristics of various policies implemented, will provide opportunities for continuous policy impact evaluation into the future.

## Appendix

**Table 8:** Name, definition, and size of SALURBAL level 3 units by country

	Level 3 (Urban)^a^	L3 (Rural)^a^	Level 3 definition	Approximate median number of households
Argentina	Radio Censal		Geographically delimited units used for census data collection.	~ 300
Brazil	Setor Censitário		Continuous area in a single urban/rural municipality equal to the workload of a census worker	~ 250
Chile	Zona Censal	Not defined	Set of blocks dividing distritos censales in urban areas	~ 700
Colombia	Sector Urbano	Not defined	Neighborhoods made up of 1 to 9 secciones urbanas	~ 350
Costa Rica	UGEB (Unidad Geostadistica Basica)		Polygon created to help with census data collection. Can be a block or other area with natural boundaries	~ 600
El Salvador	Sector Censal		Group of segmentos censales	~ 300
Guatemala	Sector Censal		Workload of a single census worker	~ 200
Mexico	AGEB		Group of blocks (manzanas)	~ 1000
Nicaragua	Sector Censal		Group of segmentos censales	~ 250
Panama	Barrio	Not defined	Sub-divisions of urban localities	~ 360
Peru	Zona Censal	Not defined	Group of adjacent blocks with physical or cultural boundaries	~ 1500

^a^Urban and rural as defined by country. In Argentina, Brazil, Costa Rica (pending confirmation), El Salvador, Guatemala, and Nicaragua (pending confirmation), the administrative units selected as L3 units cover the whole country. In Mexico, the administrative units selected as L3 units are defined to cover the whole country, but geographic files for rural areas were not available for calendar times of interest. In Chile, Colombia, Panama, and Peru, the administrative units selected as L3 units only exist in country-defined urban areas. When administrative L3 units were not defined for the whole country SALURBAL created a special SALURBAL defined L3 (a SALURBAL proxy L3). This was defined as the L2 unit minus any area covered by administrative L3 units (in the case of Mexico, Panama, and Peru). In other cases (Chile and Colombia), a smaller intermediate unit between L2 and L3 (referred to as level 2.5) was available across the country including non-urban areas. In these cases, a proxy level 3 was created by using the level 2.5 in its entirety or (in cases where the level 2.5 included areas with L3s defined) by defining the L3 proxy as the L2.5 units minus any area covered by the L3s. (Note that in Colombia, a “sector rural” is available in non-urban areas but it sometimes includes sectores urbanos, which is why the approach of treating the sector rural as a L2.5 and subtracting L3s when appropriate to create an L3 proxy had to be used)

**Table 9:** Definition of L1Metros and component subunits for each country. Component unit (L2 unit when possible or other) is italicized in each definition. Definitions and number of units are based on census data closest to 2010

Country	Metropolitan area local name or equivalent	Local definition
Argentina	Aglomerado (also known as Localidad Compuesta) *N* = 20	Agglomerations comprise one or more localities (*localidad*)—territorial divisions whose boundaries are defined by geographic characteristics or modifications of the land (i.e., buildings and streets). While agglomerations generally comprise adjacent localities, in a few cases, agglomerations include localities that are not contiguous geographically. These units are used as the sampling frame in national household surveys.
Brazil^a^	Região Metropolitanas *N* = 36 Região Integrada de Desenvolvimento Econômico (RIDE) *N* = 3	Municipalities (*municípios*) grouped together for purposes of planning and executing public actions as determined by each state. RIDE—*Municípios* that have economic ties that transcend state boundaries approved by federal legislation.
Chile	Area Metropolitana *N* = 3	Two or more *comunas* (administrative divisions of Chile similar to counties or municipalities) characterized by contiguous urban built-up areas with over 500,000 inhabitants.
Colombia	Area Metropolitana *N* = 15^b^	Two or more municipalities (*municipios*) with strong social or economic ties. The AMs have some political and administrative jurisdiction.
Costa Rica	Gran Area Metropolitana (GAM) *N* = 1	Legally created to manage urban development around San Jose. Composed of cantons (*cantones*). Some cantones in the GAM only include specific districts (*distritos*) within them.
El Salvador	Area Metropolitana *N* = 1	Legally created area post-1986 earthquake to better coordinate development across municipalities (*municipios*) of San Salvador.
Guatemala	Area Metropolitana *N* = 1	Urban agglomeration around Guatemala City that absorbs nearby populations defined by municipalities (*municipios*).
Mexico	Zona Metropolitana (ZM) *N* = 56	Two or more municipalities (*municipios*) with strong social or economic ties with a combined population of > 50,000 people, or those within the limits of one municipality with a population of > 1 million people, or those in conurbation with a US city, with a population of > 250,000 people.
Nicaragua	Region Metropolitana *N* = 1	Area of 30 municipalities (*municipios*) that are part of Managua.
Panama	Area Metropolitana N = 2	Created after the construction of the Panama Canal. It integrates the two main cities of the country (Panama and Colon). Composed of *corregimientos*.
Peru	Metropoli (also known as Area Metropolitana) *N* = 3	Population center (*centro poblado*) or group of population centers with a contiguous urban area with over 500,000 inhabitants. A population center is defined as a group of inhabitants who are linked by economic, social, cultural, or historical factors.

^a^These two types of entities for Brazil encompass different sets of non-overlapping cities

^b^This includes legally organized metropolitan areas with political administrative structure (*N* = 6) and officially recognized metropolitan areas without legally organized political administrative structures (*N* = 9, referred to as both “areas metropolitanas” and “aglomeraciones urbanas.” Population, economic, and other statistics are calculated for both types of areas by government organizations [27]

**Table 10:** Missing data and ill-defined deaths for mortality data in SALURBAL cities in 10 countries. Data corresponds to the latest available year for every country

Country	Latest year	Proportion of missing values				Ill-defined deaths
		Age	Sex	Location	Cause of death	
Argentina	2015	0.5%	0.0%	0.0%	0.0%	5.7%
Brazil	2016	0.1%	0.0%	0.3%	0.0%	5.2%
Chile	2016	0.0%	0.0%	0.0%	0.0%	2.5%
Colombia	2015	0.0%	0.0%	0.3%	0.0%	2.0%
Costa Rica	2016	0.1%	0.0%	0.0%	0.0%	3.6%
El Salvador	2014	0.1%	0.0%	0.0%	0.1%	19.4%
Guatemala	2016	0.7%	0.0%	0.0%	0.1%	8.4%
Mexico	2016	0.6%	0.1%	0.3%	0.1%	1.5%
Panama	2016	0.2%	0.0%	0.0%	0.1%	3.5%
Peru	2015	0.0%	0.0%	0.1%	0.0%	0.7%

Nicaragua mortality data is not currently available at the necessary geographic to the SALURBAL study currently

**Table 11:** Undercounting estimates and specificity of correction approaches for mortality data by country

Country	Year	National % Undercounting^a^	Correction	Source
Argentina	2013	1.3%	Blanket correction	UNDP
Brazil	2013	0%	L2 and sex-specific correction	Campos de Lima and Queiroz^b^
Chile	2013	0%	Blanket correction	UNDP
Colombia	2012	23.8%	Department, age, and sex-specific correction	DANE^c^
Costa Rica	2013	12.8%	Blanket correction	UNDP
El Salvador	2012	16.4%	Blanket correction	UNDP
Guatemala	2013	8%	Blanket correction	UNDP
Mexico	2013	− 0.8%	Blanket correction	UNDP
Panama	2013	6.8%	Blanket correction	UNDP
Peru	2013	38.3%	Department, age, and sex-specific correction	MINSA^d^

^a^National undercounting estimates come from the WHO methods and data sources for life tables 1990–2015 May 2016 update

^b^Campos de Lima EE, Queiroz BL. Evolution of the deaths registry system in Brazil: associations with changes in the mortality profile, under-registration of death counts, and ill-defined causes of death. Cadernos de Saúde Pública. 2014;30:1721–30

^c^Departamento Administrativo Nacional de Estadística, Colombia

^d^Ministry of Health of Peru http://bvs.minsa.gob.pe/local/minsa/2722.pdf

Note: Nicaragua mortality data at the necessary geographic level are not currently available to the SALURBAL study

**Table 12:** Population projections data sources and characteristics for use as denominators with mortality data.

Country	Projections years	SALURBAL level	Projections by age available	Projections age maximum	Projections by sex available	Projections by age and sex available	Projections source	Note
Argentina	2010–2015	L2	Yes	80	Yes	Yes	Local team^a^	
Brazil	2000–2015	L2	Yes	80	Yes	Yes	Local team^a^	
Chile	2002–2017	L2	Yes	80	Yes	Yes	INE^b^	
Colombia	1985-2017	L2	Yes	80	Yes	Yes	DANE^c^	
Costa Rica	2010–2017	L2	Yes	75	Yes	Yes	INEC^d^	
Guatemala	2013-2017	L2	Yes	65	Sex	Yes	MSPAS^e^	The 2008–2020 dataset was used to obtain long-term projections back to 2008. We distributed age and sex proportions according to a linear prediction using the 2013–2017 data.
	2008–2020	L2	No	N/A	No	No	OJ^f^	
El Salvador	2005–2017	L2	No	N/A	Yes	No	DIGESTYC^g^	We projected the 2015–2017 age/sex proportions back to 2010 and applied them to the 2010–2015 L2 population.
	2015–2017	L2	Yes	80	Yes	Yes	Local team^a^	
Mexico	2005, 2010, 2015	L2	Yes	100	Yes	Yes	Census^h^	We used the 2005, 2010 and 2015 census data and did a linear interpolation for the years in between, by age and sex.
Panama	2010–2017	L2	Yes	80	Yes	Yes	INEC^i^	
Peru	2005-2017	L2	Yes	80	Yes	No	INEI^j^	Data at L2 was available for age or sex, so we used the age/sex proportions at province (immediate higher level) to obtain age and sex projections at L2.
	2005–2017	Province	Yes	80	Yes	Yes	INEI^k^	

Nicaragua population projection data are not currently available at the geographic level needed by SALURBAL

^a^Records obtained from the local team are not publicly available

^b^Instituto Nacional de Estadistica, República de Chile http://ine.cl/estadisticas/demograficas-y-vitales

^c^Departamento Administrativo Nacional de Estadistica, República de Colombia. http://www.dane.gov.co/index.php/estadisticas-por-tema/demografia-y-poblacion/proyecciones-de-poblacion

^d^Instituto Nacional de Estadistica y Censos, República de Costa Rica. http://www.inec.go.cr/proyeccionpoblacion/frmproyec.aspx

^e^Departamento de Epidemiologia, Ministerio de Salud Publica, República de Guatemala. http://epidemiologia.mspas.gob.gt/index.php/dos/estadisticas-vitales/poblacion-y-proyeccion

^f^Organisimo Judicial, República de Guatemala. http://www.oj.gob.gt/estadisticaj/reportes/poblacion-total-por-municipio(1).pdf

^g^Dirección General de Estadística y Censos, República de El Salvador. http://www.digestyc.gob.sv/index.php/temas/des/ehpm/publicaciones-ehpm.html?download=517%3Aestimaciones-y-proyecciones-de-poblacion-municipal-2005-2025

^h^Instituto Nacional de Estadística y Geografía, Estados Unidos Mexicanos. http://www.beta.inegi.org.mx/default.html

^i^Contraloría General, Repúblilca de Panama. https://www.contraloria.gob.pa/inec/Publicaciones/Publicaciones.aspx?ID_SUBCATEGORIA=10&ID_PUBLICACION=556&ID_IDIOMA=1&ID_CATEGORIA=3

^j^Instituto Nacional de Estadística e Informática, República de Perú. http://proyectos.inei.gob.pe/web/biblioineipub/bancopub/Est/Lib0842/index.htm

^k^Instituto Nacional de Estadística e Informática, República de Perú http://proyectos.inei.gob.pe/web/biblioineipub/bancopub/Est/Lib1010/index.htm

**Table 13:** Health risk factor and chronic diseases surveys from SALURBAL countries used in the initial stage of harmonization

Country, survey	Sample characteristics	SALURBAL L1Admins with survey participants Median (25th—75th percentile) Sample size per L1Admin	Sampling strategy	Geographic coverage	Oversampling	Representation
Country: Argentina Survey: Encuesta Nacional de Factores de Riesgo, ENFR (National Risk Factors Survey)	Age: > 18 years *N* (2013): 32,365 Years: 2005, 2009, 2013	L1Admin: 33 Sample size: 511 (417–693)	Multistage [aglomerado censal; área (groups of radio censales); household; person 18 years or older] Stratified [population size; education level of head of household]	*Localidades* with over 5000 population	None	National, four *localidad* groups based on size, 6 regions, 23 provinces, Ciudad Autonoma de Buenos Aires, and 8 metropolitan areas > 500,000 population
Country: Brazil Survey: Pesquisa Nacional de Saúde, PNS (National Health Survey)	Age: All ages *N* (2013): 62,986 Years: 2013	L1Admin: 27 Sample size: 927 (834–1179)	Multistage [census tracts or groups of census tracts; households; person 18 years or older] Stratified [capital city, metropolitan region, or integrated economic development region, then rest of municipalities; urban/rural; total household income]	Regions (5), states or federation units (27), state capitals (27)	None	Regions (5), states or federation units (27), state capitals (27), urban and rural, metropolitan areas, and development integrated areas
Country: Chile Survey: Encuesta Nacional de Salud, ENS (National Health Survey)	Age: ≥ 15 years *N* (2010): 5434 Years: 2003, 2010	L1Admin: 19 Sample size: 85 (34–175)	Multistage [comunas; segments within comunas; household; person 15 years or older] Stratified [urban/rural with three groups of population sizes]	National	Adults ≥ 65, regions distinct to metropolitan region, rural areas	National, Regions (15), urban/rural
Country: Colombia Survey: Encuesta Nacional de Salud, ENS (National Health Survey)	Age: 0–69 years *N*: 166,474 (41,543 adults 18–29 years) Years: 2007	L1Admin: 33 Sample size: 271 (133–420)	Multistage [municipalities or combination of municipalities if small; manzanas; household; person adults 18–69 and all children 17 and under] Stratified [region; urbanization of municipal seats; urban/rural municipal population; unsatisfied basic needs]	National	None	Region, department, subregion, urban area of municipal capitals, urban/rural, by poverty level
Country: Costa Rica Survey: Encuesta Multinacional de Diabetes mellitus y Factores de Riesgo, CAMDI (Multinational Survey of Diabetes Mellitus & Risk Factors, Central American Diabetes Initiative)	Age: ≥ 20 years *N*: 1427 Year: 2005	L1Admin: 1 Sample size: 1427	Multistage [census segments; groups of households (compactos); persons within three age groups (1 selected from 20 to 39 years, 1 selected from 40 to 64 years, all selected from ≥ 65 years)]	Metropolitan San Jose	Age ≥ 65	Metropolitan San Jose
Country: El Salvador Survey: CAMDI (see Costa Rica)	Age: ≥ 20 years *N*: 1872 Year: 2004	L1Admin: 1 Sample size: 1872	Multistage [segmento censal, groups of dwellings (compacto); all household members 20 years and older]^a^	Municipio of Santa Tecla		Municipio of Santa Tecla
Country: Guatemala Survey: CAMDI (see Costa Rica)	Age: ≥ 20 years *N*: 1397 Year: 2002–2003	L1Admin: 1 Sample size: 1397	Multistage [segmento censal, groups of dwellings (compacto); all household members 20 years and older]	Villa Nueva Municipio, a part of metropolitan Guatemala City	None	Villa Nueva Municipio
Country: Nicaragua Survey: CAMDI (see Costa Rica)	Age: ≥ 20 years *N*: 1993 Year: 2003	L1Admin: 1 Sample size: 1993	Multistage [urban districts divided into 50 strata, groups of households (compacto); all family members living together 20 years and older]	Municipality of Managua	None	Municipality of Managua
Country: Mexico Survey: Encuesta Nacional de Salud y Nutricion, ENSANUT (National Survey for Health and Nutrition)	Age: all ages *N*: 96,031 (2012), 29,795 (2016) Years: 2006, 2012, 2016	2012 L1Admin: 91 Sample size: 190 (89–388) 2016 L1Admin: 59 Sample size: 39 (25–67)	Multistage [AGEB; manzana (urban) or pseudo-manzanas within localidades (rural); households; 1 person within each of the groups (0–4 years, 5–9 years, 10–19 years, 20 years and older, recent medical service user)] Stratified [socioeconomic status of AGEB at the state level]	National	AGEB with the highest index of poor socioeconomic conditions^b^	National, state, metropolitan areas, urban/rural, high/low SES
Country: Panama Survey: Encuesta Nacional de Salud y Calidad de Vida ENSCAVI (National Survey of Health and Quality of Life)	Age: ≥ 18 years *N*: 25,748 Years: 2007	L1Admin: 3 Sample size: 1773 (1738-7883)	Multistage [census segments; dwellings; persons ≥ 18 years] Stratified [indigenous population in province; urban/rural]	National	None	National, district
Country: Peru Years: Survey: Encuesta Nacional de Demografia y Salud, ENDES (National Survey of Demographics and Health)	Age: All ages *N*: 122,368 Years: 2008–2016	L1Admin: 23 Sample Size: 356 (164–629)	Multistage [conglomerado (set of census blocks –urban) or empadronamiento (set of households–rural); households; one person within each of the groups (> 15 years, females 15–49 years, children < 5 years, children < 12 years)] Stratified [department; urban/rural]	National	None	National, urban national, rural national, natural region: Lima metropolitan area, coast/mountain/jungle

^a^Documentation for El Salvador’s survey design is based on the design of other countries in the CAMDI project

^b^In Mexico, the households with the greatest deficiencies were identified through the construction of a defined social lag(rezago) index for the AGEBs; the index that was built is similar to the social lag (rezago) index built by the National Evaluation Council of the Social Development Policy for localities in 2005. https://www.coneval.org.mx/rw/resource/coneval/med_pobreza/1024.pdf
